# Assessing the Opinion of Mothers about School-Based Sexual Education in Romania, the Country with the Highest Rate of Teenage Pregnancy in Europe

**DOI:** 10.3390/medicina57080841

**Published:** 2021-08-19

**Authors:** Magdalena Iorga, Lavinia-Maria Pop, Nicoleta Gimiga, Luminița Păduraru, Smaranda Diaconescu

**Affiliations:** 1 Department of Behavioral Sciences, “Grigore T. Popa” University of Medicine and Pharmacy, 700115 Iasi, Romania; magdalena.iorga@umfiasi.ro; 2 Faculty of Psychology and Education Sciences, “Alexandru Ioan Cuza” University, 700111 Iasi, Romania; lavinia.pop@student.uaic.ro; 3 Department of Mother and Child, “Grigore T. Popa” University of Medicine and Pharmacy, 700115 Iasi, Romania; luminita.paduraru@umfiasi.ro (L.P.); smaranda.diaconescu@umfiasi.ro (S.D.); 4 “Saint Mary” Children Emergency Hospital of Iasi, 700309 Iasi, Romania; 5 “Cuza Vodă” University Maternity Hospital, 700038 Iasi, Romania

**Keywords:** teenager, sex education, sexual health, pregnancy, teenage pregnancy, sexually transmitted diseases, contraception, communication

## Abstract

*Background and Objectives:* Without mandatory school-based education, Romania is a leading European country in teen pregnancy. This survey aimed at assessing the level of knowledge and the opinions about sexual education and sexual-related issues among mothers of female teenagers aged 13–18 years old. *Material and Methods*: The survey was conducted between 2015 and 2017 and had four parts, collecting data about sociodemographic variables, the level of knowledge about sexuality, sexually transmitted diseases, and contraception. The respondents were mothers of female teenagers hospitalized in a tertiary pediatric clinic. Data were analyzed using IBM Statistical Package for Social Sciences (SPSS) Statistics for Windows, version 25 (Inc., Chicago, IL, USA). *Results*: One hundred and thirty-five mothers (42.46 ± 6.81 years old) were included in the research. Most of them were from rural areas, had graduated secondary school, were Christian-orthodox, married, and with a stable job. More than half of the mothers (61.42%) declared that they personally knew adolescents that were already mothers. In great proportion, mothers proved good knowledge about sexual education, contraception, and STDs. They considered that the minimum age for becoming married, in general, is about M = 18.62 ± 2.09 years old but in the case of their daughters, mothers appreciated that the best age would be 23.56 ± 9.37. Mothers considered that they had good communication with their daughters (M = 4.28 ± 0.99) and two-thirds sustained that they had discussed with them about sexual activity, pregnancy, sexually transmitted diseases, and contraception. In case of unwanted pregnancy of their daughters, one-third of the mothers (38.50%) would advise their girls to continue the pregnancy and 7.40% mentioned the termination of pregnancy. Two-thirds of them (74.10%) agreed to school-based sexual education. In the order of preferred sources for sexual education, mothers mentioned parents (85.90%), teachers (33.30%), and family doctors (24.40%). Comparative results regarding their own sex life and that of their daughters are presented. *Conclusions*: School-based programs should meet parental beliefs about sexuality and sexual education. School, as a creator of values and models, should find the golden ratio to better shape the personal, familial, and social needs for the healthy sexual behavior of the new generation.

## 1. Introduction

Despite the widely recognized importance of sexual health for the normal development of a person, the education programs to promote it have remained a sensitive and sometimes controversial issue. The World Health Organization (WHO) emphasized the importance of healthy sexual development to overall mental and physical well-being [1]. 

One of the main controversial ideas studied in many cultures and countries was about who is more entitled to educate children and adolescents about sexuality and healthy sexual activity. Many studies point out that parents are generally not prepared to provide complete education about sexuality. Therefore, as Shtarkshall et al. (2007) identified, health and educational systems have the obligation to provide sex education for adolescents and young adults, taking into consideration the social and familial values with respect and professionalism [2].

School-based sex education is a sensitive topic that divides opinion in all nations. A study conducted by Saarreharju et al. (2020) after the 2015 curriculum reform on sex education in Canada, for example, emphasized that the most significant topics that parents and community were interested in were children’s rights and adults’ values, political dimensions of the curriculum reform, and appropriate timing for sex education [3]. The unsuccessful attempt of the United Kingdom (UK) Government to insert sex education as a statutory part of the National Curriculum also determined many debates. Those who were willing to introduce sex education in schools were focusing on promoting the virtue of respect for every adult’s right to sexual self-determination [4]. In Japan, where sexuality education programs were implemented in junior high schools, parents appreciated that school was a more appropriate setting than home for teaching the physiological aspects of sexuality and for providing accurate information about sexual life and intimate relationship [5]. 

Many debates were developed in the United States of America (USA). For example, Gresle-Favier (2013) pointed that there was adult discrimination against children because the sex education presented abstinence-only programs [6]. In Argentina, Ambrosius (2019) showed that there was a position of domination that remained intact over the years, as children were still seen as passive subjects, unable to understand or reconstruct the social world around them [7]. Similarly, in Sweden, researchers found that parents sustained sex education in school and the delivery of diverse information, such as sexual-related risks and porn-related issues, but programs did not present information related to harm or how to avoid it [8]. 

Delivering information about sex determines a huge debate in many countries that want to implement such programs, especially because the information that is delivered to students must meet the family, community, and society’s opinions. The scientific literature about this topic is not rich. Cultural aspects, community rules, sex debates propagated by media, religious issues, parents’ reluctance, ethnicity, and ethical concerns usually limit the chances to conduct studies on children, teenagers, or caregivers. The topic was related to moral norms and parents’ consent about the proper age when sexual education must be taught but, on the other hand, the need for delivering the truth about a consensual sexual relationship, sexual criminality, sexual abuse, human sexual traffic, child marriage, teenage pregnancy, and how to recognize abuse and look for help also generated a lot of discussions among parents’ associations, non-governmental associations, and education policymakers [9]. In Sweden, parents’ attitudes regarding sex education were found to be related to the sex of the parent and the sex and age of the child. Additionally, the link between parents’ attitudes and young people’s sexual activities, especially online ones, appeared to be mediated by parental rules [10]. 

Given that mothers play an important role in the sexual education of their daughters, it is important to understand their views about sexual practice and health-related problems, as well as their opinions about the implementation of educational programs in schools. In general, formal sexuality education programming falls into one of the following two categories: abstinence-only (focusing on delaying partnered sex until marriage), and comprehensive sexuality education (discussing delayed sex until marriage, information about birth control, safe sex practices, contraception, and sexually transmitted infections). The new program models must facilitate collaboration between parents, educators, and health professionals to effectively provide sex education to young people [11].

The importance of sexual education must not be neglected since teenagers must be informed that sexual-related characteristics and sexually active life are influenced by a lot of factors. Studies [12,13,14] have proved that early menarche is related to a high body mass index, diets, poor nutrition, and low physical activity; thus, explaining these risks could increase the quality of life among teenagers. Additionally, early sexual contact is related to a high risk of pregnancy and birth, dropping out of school, and poor socio-economic outcomes and health. Married girls are ten times more likely to drop out than their unmarried peers, and pregnant teenagers are more prone to experience pregnancy and postnatal complications. Female teenagers with an early age at their first sexual act were found to be more prone to experience non-consensual sexual relationships, unwanted pregnancy, and a high frequency of sexually transmitted diseases. Because family models and parental beliefs are important factors in shaping female teenagers’ paths for education, marriage, and life, teaching sexual education in school is intrinsically related to family, community, and social norms.

Romania reported in 2011 around 34,700 teen pregnancies among females 15–19 years of age. With a pregnancy rate of 61% and a birth rate of 35%, Romania has the highest rates in Europe [15,16]. Because Romania has an increasing rate of teenage pregnancy, we wanted to identify the level of mothers’ knowledge about sexuality, and to assess their opinions about sexual programs in school. About 16 million girls 15 to 19 years old, and another one million girls under 15 give birth every year, most in low- and middle-income countries [17]. 

Few studies are focusing on sexual education in Romania, especially among teenagers. Some of the research conducted in Romania by the same extended team pointed to the socio-economic and educational factors that increased the risks for teenage pregnancy and the medical-related problems for both adolescent mothers and babies. Additionally, we proved that usually, the partner of a pregnant teenager is traditionally an older man that used age as a reason to not utilize contraceptive methods as condoms. Similarly, our previous studies showed that the younger the pregnant adolescent was, the more prone she was to become a mother again in the very next year [16,18,19]. 

The goal of the present research was to identify to what extent mothers could provide a proper sexual education for their daughters, and to find their opinions about it. We collected data about sexual-related information and practices such as the age of sexual contact, contraception, the level of knowledge about sexually transmitted diseases, the role of parents and teachers in teaching sexual education, etc. The research also evaluated mothers’ sexual behaviors, the knowledge about the daughter’s sexual life, and sexual-related information, as well as personal experiences.

## 2. Materials and Methods

This survey was developed as part of larger research with the purpose to identify the level of knowledge of hospitalized female patients’ mothers registered in Saint Mary University Hospital for Children about sexually transmitted diseases, the risk of pregnancy, methods of contraceptives, and to assess attitudes and behaviors related to sexual activity among daughters.

### 2.1. Participants and Data Collection

The survey was distributed between September 2016 and November 2017 among mothers who visited their daughters while they were hospitalized in a pediatric tertiary center. The caregivers were informed about the purpose of the research, and they were assured about the confidentiality of data. No incentives were offered to the voluntary respondents. Informed consent was previously obtained from the mothers. The inclusion criteria were parents that had at least one female teenager (aged 13–18 years) hospitalized during the considered period, agreeing to sign the informed consent. Exclusion criteria: mothers aged under 18, mothers of pregnant teenagers, mothers of normal children (with no psychological or psychiatric diagnostic, no medical disease that impacted physical growth and sexuality, such as genetic disorders).

The survey was delivered to all one hundred and fifty-two mothers who visited their daughters during hospitalization. The response rate was 95%. We excluded ten questionnaires because the respondents filled them in, but the informed consent was not signed. Finally, one hundred and thirty-five forms were included in our databases and considered for the present research. 

### 2.2. Instruments

A questionnaire was constructed in purpose for this research and contained four parts:

- The first part collected sociodemographic data such as age, gender, religion, number of children, marital status, level of education, environment, monthly income, alcohol abuse or physical abuse, and information about the relationships between the family members.
- The second part gathered information about sexual-related data and sexual life such as the age at which they had their first menstruation, the age at which they had their first sexual contact, from whom they received the first information about menstruation, if they knew the term sex education, if they knew the term contraception, and if they used contraception, as well as if they had information about diseases with sexual transmission.
- The third part contained items to identify the extent to which mothers considered themselves informed about their daughters’ lives and sexual activity and whether they had begun sexual activity. The questions focused on whether mothers knew that girls had/did not have a partner, whether girls already had menstruation, whether they had told them about menstruation, who would like to deliver sex education to their daughters, whether they had talked to their daughters about sexual activity, pregnancy, sexually transmitted diseases, or methods of contraception.
- The fourth part of the questionnaire had items that wanted to identify the mothers’ opinions about specific situations, such as what advice they would give to their daughters in case they became pregnant, what contraceptive methods would be appropriate for their daughters to use, if they would agree that their daughters should have sex before marriage, at what age they would like their daughters to marry and at what age they considered as appropriate for their daughters to have their first child.

### 2.3. Statistical Analysis

All analyses were performed using the IBM Statistical Package for Social Sciences (SPSS) Statistics for Windows, version 25 (Inc., Chicago, IL, USA). The descriptive statistics of socio-demographic data were expressed as means and standard deviations (SD), frequencies and percentages. The Mann–Whitney test was performed for the comparative analysis among categories. The correlation analysis was performed using the Spearman correlations. The level of statistical significance was set at *p* < 0.05.

### 2.4. Ethical Approval

The present study was conducted in accordance with the Declaration of Helsinki and the protocol was approved by the Ethical Committee of Saint Mary Emergency Hospital for Children, number 23403/30.10.2015, as part of a larger study supported by the European Society of Contraception and Reproductive Health. The project was entitled “Identifying and tackling economic, cultural, and social factors that lead to inappropriate sexual education in Romanian teens” (P-2015-A-05). Informed consent was obtained from all mothers included in the research.

## 3. Results

Sociodemographic data and medical characteristics were collected. Additionally, the answers for items regarding mothers’ opinions were analyzed. 

### 3.1. Sociodemographic and Family-Related Data

Sociodemographic data were gathered, together with the financial status, environment characteristics, and family-related information. All questioned participants were mothers aged 42.46 ± 6.81 (minimum of 29, maximum of 55 years old), most of them being married or in a relationship, Christian-orthodox, and coming from rural areas. 

Detailed results about marital status, level of education, number of children, employment, and the presence of adverse experiences such as alcohol consumption or physical abuse between partners are presented in Table 1.

### 3.2. Level of Knowledge about Sexually Transmitted Diseases, Sexual Education, and Contraception

A series of items were formulated to identify the level of knowledge about sexually transmitted diseases, healthy sexual behaviors, preventive measures, and sexual activity.

Most mothers had knowledge about sexually transmitted diseases (83.08%). They were also asked to declare which STD they knew and how they can be transmitted. The analysis of data revealed that mothers had knowledge about the following STDs: syphilis (61.54%), HIV (80.77%), trichomoniasis (8.46%), candidosis (25.38%), gonorrhea (20.77%), B Hepatitis (43.08%), and chlamydia (7.69%).

The analysis of data showed that more than two-thirds of the respondents (86.7%) knew what the term sex education meant and what it referred to, what contraception was (84.40%), and that they knew contraceptive methods (86.70%).

One item investigated how many contraceptive methods they knew, and 74.10% mentioned oral contraceptives, 66.70% condoms, and 43% mentioned as a contraceptive method “the use of calendar”.

More than half of the mothers (61.42%) declared that they personally knew adolescents that were already mothers. Additionally, we identified that the minimum age that mothers considered proper for marriage was M = 18.62 ± 2.09 years old.

### 3.3. Medical and Sexual Related Characteristics of Mothers

Mothers were asked about the first age at which the menstruation occurred and at what age they had their first sexual intercourse. Data about the age of marriage, when they became mothers for the first time, or who were the sources of information (mothers, siblings, teachers, friends) were also collected. Detailed results are presented in Table 2.

### 3.4. The Mother–Daughter Relationship and the Impact on Sexual Education

The mothers were asked to rate, on a five point Likert-like scale (from 1—*extremely poor*—to 5—*extremely good*), their satisfaction concerning the communication with their daughters. The statistical analysis proved a score of M = 4.28 ± 0.99, meaning that mothers appreciated that they had good communication with their offspring. Detailed results are presented in Table 3.

A series of items were formulated to investigate what kind of contraception methods they counselled their daughters to use. The most frequent mentioned contraceptives methods were condoms (36.30%) and oral pills (34.80%). A smaller percentage mentioned “the use of calendar” (8.10%) and a smaller percentage, about 7.40%, considered that the partner must take precautions to not impregnate the teenage girl.

In case of unwanted pregnancy, one-third of the mothers (38.50%) would advise their girls to continue the pregnancy, or let the adolescents choose for themselves (28.10%), 11.90% considered that in case of pregnancy their daughters must marry, 7.40% would prefer that their offspring choose termination of the pregnancy, and 1.50% mentioned that probably the girl would be sent to live with her partner. A total of 12.60% of questioned mothers mentioned that they had no idea what to tell their daughters.

The analysis of answers proved that mothers appreciated that the most frequent risks related to teen pregnancy were school dropout (68.30%) and psychological trauma (59.20%).

### 3.5. The Opinion about School-Based Sexual Education

Mothers were asked if they would like their daughters to have sex education classes at school: 74.10% agreed that their daughters had sexual education in school, 10.40% disagreed with it, 12.6% sustained that they did not know, and 3% mentioned that it was too early for their daughters to have classes about sexual education.

One item investigated mothers’ opinion about who would be more appropriate to teach sexual education. The frequency of answers showed that the more preferred sources were parents, teachers, and family doctors. Detailed results are presented in Table 4.

There were significant differences in the level of education of mothers regarding certain items. Mothers who had low levels of education, namely, lower secondary school, had on average a higher number of children (Mdn = 3) than mothers who had a university level of education (Mdn = 2), *p* = 0.005. In addition, mothers with a lower level of education married earlier and had their first child at a younger age as opposed to participants who reported a higher level of education. Family income was considerably higher for mothers with a higher level of education, and the number of methods of contraception was better known among mothers with a higher level of education. The detailed results for these items are presented in Table 5.

The correlational analysis showed that there were significant associations between the age at which mothers gave birth to their first child and certain variables. We identified that the more mothers gave birth to younger children, the higher the number of children in the family (r = −0.395 **, *p* < 0.001). The age at which mothers gave birth to their first child was positively correlated with the onset of menarche, in the sense that the higher the age of onset of the first menstruation, the higher the age at which mothers give birth to the first child (r = 0.217 *, *p* = 0.029). At the same time, the correlational analysis showed that the older the age at which mothers wanted their daughters to marry, the higher the age at which mothers wanted their daughters to become pregnant with their first child (r = 0.824 **, *p* < 0.001).

The results showed that the younger the mothers, the lower the level of education, the age of the first sexual contact, the age at which mothers married and gave birth to their first child were. Negative correlations existed between mothers’ ages and the level of education (r = −0.270 *, *p* = 0.049), the age of first sexual intercourse (r = −0.518 **, *p* < 0.001), the age at which they married (r = −0.479 **, *p* < 0.001) and the age at which they had their first child (r = −0.562 **, *p* < 0.001). The level of education correlated positively with the mothers’ age of marriage (r = 0.629 **, *p* < 0.001) and the age at which they gave birth to the first child (r = 0.640 **, *p* < 0.001). The analysis of data also revealed that the more educated the mothers were, the more they would marry and give birth to the first child later.

The family income correlated positively with the mother’s level of education (r = 0.625 **, *p* < 0.001), the age at which the mothers married (r = 0.419 **, *p* < 0.001), and the age at which they gave birth to the first child (r = 0.494 **, *p* < 0.001), in the sense that the higher the family income, the higher the level of education and the age at which the mothers married at, respectively, gave birth to the first child. At the same time, family income correlated negatively with the number of children in the household because the higher the family income, the lower the number of children in the household (r = −0.357 **, *p* < 0.001).

## 4. Discussion

Numerous studies have pointed to the need for sexual education among children and teenagers and identified that schools and parents were the most important sources of knowledge. Many countries have implemented sexual education programs. Education for sex and sexuality was proved to positively impact adolescents’ knowledge about sexual activity, safe sex behavior, contraception, and how to avoid sexual abuse, teenage pregnancy, sexually transmitted diseases, and risk for pornography and bullying. Healthy sexual activity has a great impact on physical, psychological, and social status [2,8,9].

There are many educators regarding sexuality and sexual activity: family, school, church, media, society, peers, etc. The results of the present study are important to shape the context of future school-based programs in Romania. Different results were also pointed out by the scientific literature, and they are diverse and contradictory. For example, a study conducted in Israel identified that most of both male and female students from secondary schools mentioned school as their preferred source of sex education and evaluated home as their last choice. The same study identified that only one-quarter of teenagers wanted parents to be their primary source of information regarding sexuality and sex life [20]. A study developed in the United Kingdom suggested that one-third of the adolescents preferred to receive additional information about sex from parents (33%) and schools (34%) [21].

In general, the scientific literature focused on the opinion of parents about sexuality and sex education in family and school. Diverse studies were conducted in different countries where this topic is a taboo or included a different population where sex education was limited by the national curricula. For example, Iranian parents considered that sexual health-related information must be trained through life and practice and, therefore, education in school is not necessary. The questioned parents stated that sexual health education encouraged children to be rude [22]. In opposition with these results, Reza et al. (2020) pointed out that Indonesian parents agreed that children must be trained by knowing parts of the body that may or may not be touched by others, covering up the genitals, and acknowledging who can hug and kiss the cheek of a child, knowing the abusive behavior and how to reject and report it [23]. Nair et al. (2012) conducted a study on Indian parents. The authors identified that more than half of them were not sure whether information on various reproductive and sexual health topics should be given to adolescents and were not sure about the way to inform their offspring about it [24]. McKibben (2017) identified that sex education in the UK must take into consideration gender risks. The author identified that targeting grooming and problematic sexual behavior is mandatory in children and adolescents, and formal content of the sex education must focus on sexual health, normal sexual development, mutual consent, the minimum age for sexual contact, gender stereotypes, grooming and sexual abuse, pornography, and healthy sexual life [25]. The same ideas were developed by Green and Mason (2002) who also found that girls conflated love and sex, whereas boys constructed sex as a physical conquest. This type of construction made girls extremely vulnerable to sexual exploitation, especially female adolescents with no families to support them or those living in residential children’s homes [26].

Jankovic et al. (2013) identified that most Croatian parents found it inappropriate to be talked to about sexual satisfaction and pleasure, masturbation, pornography, and prostitution [27]. On the other hand, Baker (2016) showed that British teachers and professionals in the UK declared that since the Internet made sexually explicit information more accessible to young people, this could have a negative impact on their sexual development and future relationships during their lifetime. The study revealed that young people and teachers considered that there were numerous negative effects of viewing pornography, especially online, and this topic must be discussed during classes at a young age [28].

Some authors counselled that the precise age at which this information should be provided is closely related to the physical, emotional, and intellectual development of the child. Some studies identified that the proper age was differently mentioned by mothers. For example, more than 60% of Nigerian women considered that children had to be informed about sexual-related issues before the age of ten [29].

Opara et al. (2010) identified that whereas almost half of the mothers found out about menstruation from their mothers, only thirteen (8.2%) of the questioned mothers discussed menstruation with their daughters. Educated mothers proved to be more aware of the importance of sexual education among offspring and were more willing to communicate with their children about sexual activity, contraception, and sexually transmitted diseases [29]. Kose et al. (2014) identified significant correlations between the educational status of the mothers and their knowledge about HPV vaccination but no significant correlation in terms of economic conditions [30]. Similar results were provided by a study conducted in nine European countries and revealed that maternal support and the maternal level of knowledge about sexuality are negatively correlated to the early initiation [31]. Genz et al. (2017) identified that 89.2% of the teenage girls were able to properly define the concept of a sexually transmitted disease [32] and Doreto et al. (2007) showed that teenagers knew of five to six sexually transmitted diseases, such as syphilis (35.6%), genital herpes (33.3%), gonorrhea (30.0%), and HPV (27.2%) [33]. Early pregnancy was related to an older partner [18] less interested in contraception. The evidence suggested that much of the adolescent sexual activity is spontaneous, unplanned, and sometimes involuntary [34], and that the most common profile of patients looking for care for sexually transmitted diseases is men with a high level of education, poor use of condoms, and a high number of partners [35].

Mothers’ age at which menarche occurred was about 13.50 ± 1.64 and in what their daughters were concerned, the respondents mentioned 12.47 ± 1.29 years old. Our results are congruent with those from the scientific literature. Menarche occurs between the ages of 10 and 16 years in most girls in developed countries. Over the past century, the age at menarche has fallen in industrialized countries, but that trend has stopped and may even be reversing. In 1840, the average age at menarche was 16.5 years but nowadays it is 13 [36]. The same trend was registered in many studies from different countries: in Norway, Gottschalk et al. (2020) developed a study on an extremely large population of women and showed that the mean age at menarche was 13.42 years among women born during 1936–1939, and 13.24 years (95%) among women born during 1960–1964 [37]. A study conducted by Biro et al. (2018) on American female teenagers identified that the median age at menarche overall was 12.25 year [38]. Meng et al. (2017) also found a continuous downward secular trend of age at menarche for Chinese girls in both urban and rural areas, who were born from 1973 to 2004 [39].

Our research showed that the age mothers married at was 21.27 ± 3.51. They also appreciated that the minimum age for marriage should be about M = 18.62 ± 2.09 years old, but in the case of their daughters, the statistical analysis proved that they would prefer an older age (23.56 ± 9.37). Therefore, compared to their own experience, mothers would like their daughters to marry later than they did.

Our survey showed that mothers appreciated that the most frequent risks related to teen pregnancy were school dropout (68.30%) and psychological trauma (59.20%), but a lot of researchers identified that there was a high risk for physical health as for psychological health among teenage mothers. For example, Paul (2018) proved that an early maternal age at marriage substantially increased the risk of adverse pregnancy outcomes (pregnancy and post-natal complications) [40]. These results strongly suggested that the existing laws and policies must be strengthened to decrease the age at pregnancy or marriage among teenagers [41].

We identified that more than two-thirds of the mothers considered that school-based sexual education was useful for their daughters but also suggested that the main sources for education were parents, teachers, and family doctors. Our results are congruent with those from other countries. Shams et al. (2017) conducted a study on Iranian mothers and showed that the respondents believed that limited sexual health education for adolescent girls was necessary. The authors stated that mothers must be trained how to deliver sexual education to their daughters and identified that trained mothers were best equipped to educate their daughters. Special training on communication, sexual health education, and how to develop a good relationship between parent and child were appreciated as being the key to empowering mothers to be ready to educate their daughters regarding sexual life and to answer their children’s questions regarding sexual issues at any age [21]. Lefkowitz et al. (2000) showed that a trained mother talked more freely about sexuality to their daughters and acted less judgmental [42].

### 4.1. Reflections and Planning

The opinion about sexual education is influenced by familial, social, and community norms, peers’ influences, religious beliefs, or own experience—the relationship with parents, especially mothers, the level of education for both teenagers and parents, the relationship with the partner (mutual consent or abuse); sexual education must take into consideration a lot of factors. School and family are the most secure environments and many policymakers had to tailor the program for teenagers to satisfy the triad adolescent–family–school. The results of the present study highlighted this idea, investigating mothers from both hypostases: mothers of teenage daughters and those of former teenage daughters.

### 4.2. Strengths and Limitations of the Study

The most important strength of the study was that the results covered a great gap of scientific information related to the impact of sexual education on adolescent’s life and the opinions of mothers, as one of the main important sources of education for girls.

The second strength was due to the study population; Romanian teenagers are among the high-risk populations with continuously increasing rates of unwanted pregnancies and births in girls under the age of 18. Therefore, the present paper focused on what mothers know about their daughters’ sexual lives and how they can help them.

Our study presented some limitations. First, the research was conducted in a single-center hospital for children. Even if the University hospital gathers patients from almost one-quarter of the country and the results can be generalized for the Romanian teenage population, it must be treated with precociousness by other countries because it was already proved by the scientific literature that national laws, community rules, and religious aspects must be always taken into consideration when collecting data about sexual activity. Secondly, the results obtained were specific for the Romanian population (considering that race is related to different ages at menarche, for example) or that the present study did not take into consideration other variables (such as high body mass index, poor nutrition, low physical activity or a low financial status that proved to be related to earlier menarche). Additionally, only mothers who had a comfortable literacy level agreed to fill in the questionnaire, limiting the investigated population.

## 5. Conclusions

A school-based program should rely on parental beliefs and the family–adolescent relationship, considering that parental sexual education and school-based sexual education are not congruent. That is why school, as a creator of values and models, must find the golden ratio to satisfy family, community, and social norms, and to preserve a healthy physical and psychological healthy generation.

## Figures and Tables

**Table 1:** Sociodemographic characteristics ^1^.

Sociodemographic Characteristics	%/M ± SD
Age	42.46 ± 6.81
Environment	
Urban	36.60
Rural	63.40
Religion	
Christian-orthodox	92.50
Catholic	4.50
Pentecostal	3.00
Level of education	
Primary school	4.50
Secondary school	42.20
High school	39.80
University	12.50
Nationality/Ethnicity	
Romanian	97.00
Roma	2.30
Others	0.70
Number of children	
One child	20.70
Two children	37.00
Three children	18.50
Four children	3.70
Five children	5.20
Six children	5.20
More than six	9.70
Marital status	
Married/in relationship	80.92
Single/divorced/widow	19.08
Employment	
Employed (stable job)	50.81
Unemployed (temporary without job)	18.55
Housewife	30.65
Partner working abroad	20.10
Partner is an alcoholic	10.69
Partner is a physical aggressor	1.55

^1^ Number of answers (N) and percentage (%); means and standard deviations (M ± SD).

**Table 2:** Sexual-related information data.

Items	M ± SD ^1^
Age at which menstruation occurred	13.50 ± 1.64
Age of first sexual contact	19.50 ± 2.76
Age at which they married	21.27 ± 3.51
Age at which they had their first child	22.23 ± 4.71
From whom they received the first information about menstruation	
Parents	75.63
Siblings	6.72
Friends	7.54
Teachers/school staff	6.72
Others	3.39

^1^ Means and standard deviations (M ± SD).

**Table 3:** Items focusing on mothers’ level of knowledge about their daughters’ sexuality and sexual activity.

Items	M ± SD ^1^
At what age did your daughter have her first period?	12.47 ± 1.29
At what age did you tell your daughter about menstruation?	10.77 ± 2.61
Did you talk to your daughter about sexual activity, pregnancy, STD or contraception?	
Yes	72.40
Is your daughter sexually active?	
Yes	14.10
No	74.10
I do not know	11.80
Does your daughter have a partner?	
Yes	37.80
No	55.60
I do not know	6.60
Has your daughter ever become pregnant?	
Yes	3.70
No	93.30
I do not know	3.00
If your daughter became pregnant, would you share it with your spouse?	
Yes	84.40
No	0.70
I do not know	14.70
Among your daughter’s friends, are there teenagers who became pregnant?	
Yes	20.00
No	54.80
I do not know	25.20
Do you agree that your daughter should have sex before marriage?	
Yes	31.10
No	37.10
I do not know	31.80
At what age would you like your daughter to marry?	23.56 ± 9.37
At what age would you like your daughter to have her first child?	24.83 ± 3.06

^1^ Percentages (%); means and standard deviations (M ± SD).

**Table 4:** The frequency of answers to the item who do you think should teach your daughter about sex education ^1^.

Who Do You Think Should Teach Your Daughter about Sex Education?	Yes (%)
Parents	85.90
Teachers/school staff	33.30
Friends	1.50
Siblings	5.09
Priest	3.70
Family doctor	24.40

^1^ Percentages (%).

**Table 5:** Comparative results considering the level of education ^1^.

Items	Mann–Whitney U	Z	*p*	Secondary School	University Studies
The number of children you had	226.500	−2.836	0.005	3	2
At what age did you marry?	64.500	−5.069	0.000	19	24
At what age did you have your first child?	56.000	−5.238	0.000	20	24.5
The family’s monthly income	71.000	−4.960	0.000	1	5
What methods of contraception did you know?	145.000	−4.112	0.000	2	3

^1^ Median, Mann–Whitney results.

## Data Availability

Data available upon request from corresponding author.
